# Machine learning-assisted substrate binding pocket engineering based on structural information

**DOI:** 10.1093/bib/bbae381

**Published:** 2024-08-05

**Authors:** Xinglong Wang, Kangjie Xu, Xuan Zeng, Kai Linghu, Beichen Zhao, Shangyang Yu, Kun Wang, Shuyao Yu, Xinyi Zhao, Weizhu Zeng, Kai Wang, Jingwen Zhou

**Affiliations:** School of Food Science and Technology, Jiangnan University, 1800 Lihu Road, Wuxi, Jiangsu 214122, China; Engineering Research Center of Ministry of Education on Food Synthetic Biotechnology and School of Biotechnology, Jiangnan University, 1800 Lihu Road, Wuxi, Jiangsu 214122, China; Science Center for Future Foods, Jiangnan University, 1800 Lihu Road, Wuxi, Jiangsu 214122, China; Science Center for Future Foods, Jiangnan University, 1800 Lihu Road, Wuxi, Jiangsu 214122, China; Key Laboratory of Advanced Process Control for Light Industry (Ministry of Education), School of Internet of Things Engineering, Jiangnan University, 1800 Lihu Road, Wuxi, Jiangsu 214122, China; Science Center for Future Foods, Jiangnan University, 1800 Lihu Road, Wuxi, Jiangsu 214122, China; Science Center for Future Foods, Jiangnan University, 1800 Lihu Road, Wuxi, Jiangsu 214122, China; Science Center for Future Foods, Jiangnan University, 1800 Lihu Road, Wuxi, Jiangsu 214122, China; Science Center for Future Foods, Jiangnan University, 1800 Lihu Road, Wuxi, Jiangsu 214122, China; Science Center for Future Foods, Jiangnan University, 1800 Lihu Road, Wuxi, Jiangsu 214122, China; Science Center for Future Foods, Jiangnan University, 1800 Lihu Road, Wuxi, Jiangsu 214122, China; Science Center for Future Foods, Jiangnan University, 1800 Lihu Road, Wuxi, Jiangsu 214122, China; Key Laboratory of Advanced Process Control for Light Industry (Ministry of Education), School of Internet of Things Engineering, Jiangnan University, 1800 Lihu Road, Wuxi, Jiangsu 214122, China; Engineering Research Center of Ministry of Education on Food Synthetic Biotechnology and School of Biotechnology, Jiangnan University, 1800 Lihu Road, Wuxi, Jiangsu 214122, China; Science Center for Future Foods, Jiangnan University, 1800 Lihu Road, Wuxi, Jiangsu 214122, China; Jiangsu Province Engineering Research Center of Food Synthetic Biotechnology, Jiangnan University, 1800 Lihu Road, Wuxi, Jiangsu 214122, China

**Keywords:** substrate binding sites, deep learning, acid phosphatase, proline 4-hydroxylase

## Abstract

Engineering enzyme–substrate binding pockets is the most efficient approach for modifying catalytic activity, but is limited if the substrate binding sites are indistinct. Here, we developed a 3D convolutional neural network for predicting protein–ligand binding sites. The network was integrated by DenseNet, UNet, and self-attention for extracting features and recovering sample size. We attempted to enlarge the dataset by data augmentation, and the model achieved success rates of 48.4%, 35.5%, and 43.6% at a precision of ≥50% and 52%, 47.6%, and 58.1%. The distance of predicted and real center is ≤4 Å, which is based on SC6K, COACH420, and BU48 validation datasets. The substrate binding sites of *Klebsiella variicola* acid phosphatase (KvAP) and *Bacillus anthracis* proline 4-hydroxylase (BaP4H) were predicted using DUnet, showing high competitive performance of 53.8% and 56% of the predicted binding sites that critically affected the catalysis of KvAP and BaP4H. Virtual saturation mutagenesis was applied based on the predicted binding sites of KvAP, and the top-ranked 10 single mutations contributed to stronger enzyme–substrate binding varied while the predicted sites were different. The advantage of DUnet for predicting key residues responsible for enzyme activity further promoted the success rate of virtual mutagenesis. This study highlighted the significance of correctly predicting key binding sites for enzyme engineering.

## Graphical Abstract

**Graphical Abstract:**
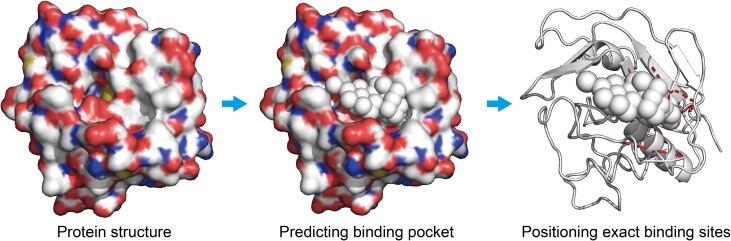

## Introduction

Structure-based protein design has revolutionized the field of protein engineering [1, 2]. The conventional steps in enzyme activity engineering include protein structure acquisition [3, 4], substrate binding sites prediction, and evaluating mutagenesis energy [5]. Acquisition of protein 3D structure is difficult owing to the labor-intensive crystallization or Cryo-electron microscopy (Cryo-SEM) procedure, and significant progress has been made in protein modeling [3, 6–8]. The protein modeling tools essentially support downstream processes such as molecular docking and virtual mutagenesis, and can be used in various combinations to discover drugs and functional proteins [9, 10]. However, determination of substrate binding sites requires extensive experimental work to validate the key sites [11]. As the molecular binding of protein–ligand and enzyme–substrate can critically affect the specificity and activity of protein/enzyme functions [12, 13], it is crucial to develop a tool for rapid and accurate detection of substrate binding sites for target enzymes.

Previous studies employed template-, geometric-, or probe-based information to guide the prediction of ligand/substrate binding sites [14–18]. Template-based methods require enriched databases covering annotated protein–ligand binding information [14]. Geometric-based methods scan the cavities of the protein structure to predict the possibility of each cavity based on Voronoi tessellation or α-spheres [15]. Probe-based methods are established on binding energy and used virtual probes to score the cavities [16]. Deep learning (DL) techniques of convolutional neural network (CNN) have been noted to exhibit great performance in segmenting protein–ligand binding sites in a 3D image [19–21]. Although DL-based models can directly learn the binding behaviors from the contact between protein and ligand, the obtained success rate (SR) was still not satisfied [19–21]. Meanwhile, it is crucial to further investigate the significance of the predicted key sites based on experiments.

The network architecture and training datasets can contribute to model accuracy [22, 23], and the utility of a deeper network can assist for capturing target features [24]. From Kalasanty to PUResNet, the utility of ResNet for deeply extracting the protein–ligand features obtained improved accuracy for predicting ligand binding sites [20]. In particular, DenseNet, a more recently developed network, has been used to address the limitation of ResNet [25]. DenseNet can extract the features of 3D targets and has been successfully implemented to protein-based tasks [26]. Besides, the annotated protein–ligand information based on protein sequences is higher than that of protein structure, supporting the use of sequential-based networks [27]. Therefore, selecting a proper strategy for enlarging a 3D structural dataset may further benefit the correlated training [28].

**Figure 1:**
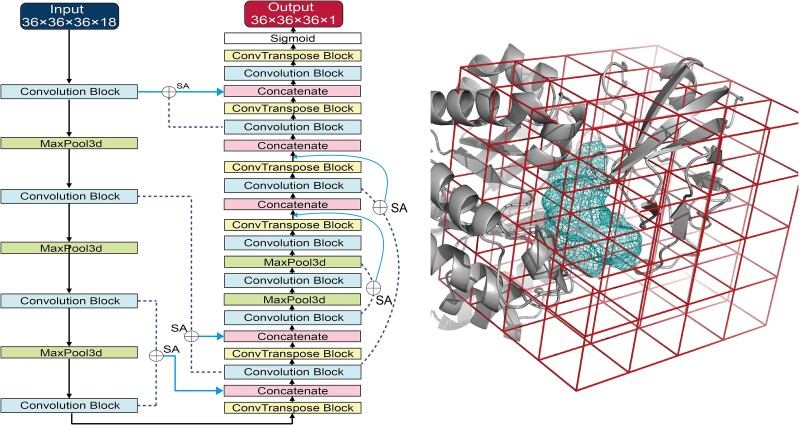
Architecture of DUnet for protein/enzyme binding sites prediction. Architecture of DUnet for protein–ligand binding sites prediction. The protein structure was prepared as a 3D image with a size of 36 × 36 × 36, and 18 features were used to describe the atomic characteristics of the protein. The network integrated DenseNet, UNet, and self-attention for feature extraction and image size recovery, respectively, the output image size was 36 × 36 × 36, and only one feature was used to describe if the atom was the binding site or not. Convolution block comprised of Conv3d and BatchNorm3d, ConvTranspose block comprised of ConvTranspose3d, and SA represented self-attention, which learned features from the two given objects (shown by dashed line), through concatenation to combine the features from the upper block. A steric representation of protein and the predicted binding sites (shown as density) was shown on the right.

To adapt a much deeper network for feature extraction, we developed a 3D structure-based substrate binding site prediction model named DUnet (Fig. 1), which established on the DenseNet [25], self-attention (SA) [29], and UNet. The size of the training set was enlarged using the data augmentation method [20, 30]. Initial assessment of DUnet for identification of key binding sites for enzyme engineering was conducted using *Klebsiella variicola* acid phosphatase (KvAP) and *Bacillus anthracis* proline 4-hydroxylase (BaP4H) [31]. Virtual saturation mutagenesis was carried out based on the predicted binding sites, and we experimentally validated the top-ranked 10 single mutations to illustrate that the varied predicted binding sites can affect the efficiency of virtual mutagenesis. We also provided a combined script by predicting the binding sites using DUnet followed by accommodating substrate for molecular docking [32]. Finally, DUnet was used to predict the ligand binding sites of Swiss-Prot annotated proteins.

## Results

### Model architecture and training set affected model performance

Predicting protein–ligand binding sites was treated as 3D image segmentation problem. Therefore, the network developed in this study was all UNet-based, to learn features through the encoder part and then recover the size by the decoder part. Previous study showed that architecting a deeper network can benefit model performance [25]; thus, we directly attempted DenseNet and ResNet for feature extraction. Meanwhile, to address the limitation of CNN focused on extracting local features, we integrated SA [29] for extracting information from the subspaces [33]. Training the network using the enlarged dataset by rotating the structure samples within the scPDB_5020 dataset (5020 samples) by 15° can improve model accuracy (Table S1). Therefore, the networks were trained using the enlarged dataset (10 040 samples). The performance of the models was evaluated using two metrics: SR with precision ≥50% (SR-PRE) and SR with the predicted and actual pocket distance center-center (DCC) ≤ 4 Å (SR-DCC) [20, 21]. The precision is obtained by:


$$ \mathrm{Precision}=\frac{\mathrm{Correctly}\ \mathrm{predicted}\ \mathrm{binding}\ \mathrm{sites}\ \left(\mathrm{TP}\right)}{\mathrm{TP}+\mathrm{Incorrectly}\ \mathrm{predicted}\ \mathrm{binding}\ \mathrm{sites}\ \left(\mathrm{FP}\right)} $$

The models with different network architectures were trained model and validated based on three validation sets, including SC6K (2417 samples), COACH420 (296 samples), and BU48 (62 samples) [20]. DUnet obtained the highest SR-PRE of 48.4% and 35.5% on SC6K and COACH420, while the simply DenseNet-based network obtained the highest value on BU48 validation sets. Meanwhile, DUnet also exhibited the highest value of SR-DCC of 52% and 47.6% on SC6K and COACH420, and DenseNet-based network displayed the highest value on BU48. These results showed the DenseNet-based network with higher capacity for predicting the binding sites, and implementing self-attention to the simply DenseNet- and ResNet-based network further promoted their overall performance.

### Comparison to the deep learning–based methods

**Figure 2:**
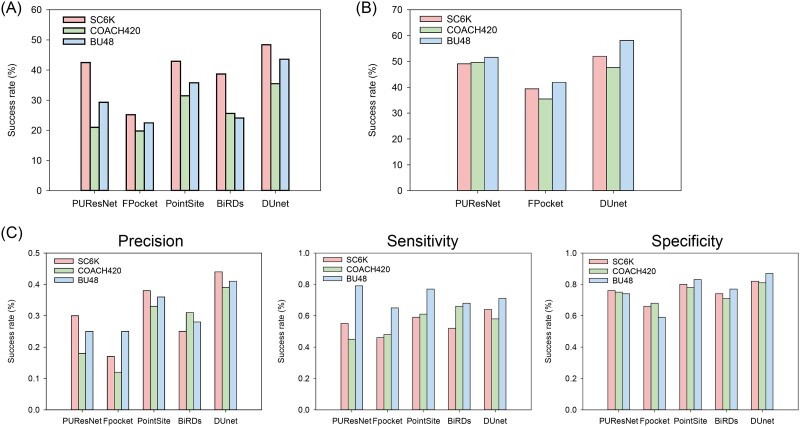
Comparison of DL-based methods. SR of precision ≥50% (A) and DCC ≤ 4 Å (B) and (C) the average precision, sensitivity, and specificity while these DL-based methods predicted at least one pocket. Noted that the output of PointSite and BiRDs were binding sites within proteins and differ from the calculated density of where ligand exists, we thus did not calculate the SR-DCC of the two methods.

To confirm the performance of DUnet, a systematical comparison of DUnet with other competing DL-based methods was conducted. The comparison was performed with 3D structure-based methods including PUResNet, PointSite, and Fpocket and sequence-based method BiRDs. As shown in Figure 2A, DUnet outperformed the aligned four methods for correctly predicting the real binding sites, displaying 5.9%, 4%, and 7.8% of SR-PRE higher than the second best methods, respectively. These results indicated that DUnet can dramatically improve the accuracy for predicting the exact binding residues, avoiding prediction of excessive larger pocket-covered unbound residues.

DCC analysis was used to evaluate the center of predicted and real binding pockets, but note that the real pockets within the validation sets of COACH420 and BU48 were shown by real ligands and, to some extent, not fully represent the whole binding sites. As shown in Figure 2B, DUnet exhibited the highest SR-DCC on SC6K and BU48, displaying 2.9% and 6.5% higher than the second best methods. PUResNet displayed the highest SR-DCC on COACH420, displaying 2% higher than DUnet. Combining the overall DCC results regarding the sample amount, DUnet is comparably accurate for predicting the real center of the binding pocket.

In addition, the evaluation metrics by calculating the average precision, sensitivity, and specificity based on the results with predicted pockets were used to further compare these methods. The sensitivity and specificity are obtained by:


$$ \mathrm{Sensitivity}=\frac{\mathrm{TP}}{\mathrm{TP}+\mathrm{FN}} $$

$$ \mathrm{Specificity}=\frac{\mathrm{TN}}{\mathrm{TN}+\mathrm{FP}} $$

As shown in Figure 2C, the precision of DUnet displayed the highest values for all the three test sets. DUnet, BiRDs, and PUResNet obtained the highest sensitivity on SC6K, COACH420, and BU48, respectively. For specificity, DUnet displayed the highest values for SC6K and BU48, while PointSite displayed the highest on COACH420. These results indicated that the methods including BiRDs and PUResNet were able to predict correct residues to the utmost extent, while DUnet was able to find the correct residues and avoid false-positive residues.

### Blind test

Even though the aligned DL methods were trained and validated using the same datasets, there are still samples with high similarity above 40% within the training and validation set. Thus, we used a curated CAMEO validation set, which, with similarity of lower than 25%, aligned with the training set used in this study for further evaluating the performance of these methods [34, 35]. The SR-PRE obtained by the DL methods was used based on the 81 valid entries. As shown in Table 1, DUnet achieved the highest SR-PRE, while with minor improvement compared with PointSite, but the given two methods were more than 4.9% and higher than the other methods. Meanwhile, the precision and specificity values obtained by using DUnet were the highest, and using PointSite achieved the highest sensitivity value (Table 1). These results confirmed the capability of DUnet for predicting the binding sites for novel structures with low similarity to the training samples.

**Table 1:** Performance of DL-methods on CAMEO validation set

DL-methods	SR-PRE	Precision	Sensitivity	Specificity
PUResNet	41.4%	0.28	0.54	0.76
Fpocket	26.5%	0.17	0.51	0.68
PointSite	46.3%	0.38	**0.65**	0.79
BiRDs	30.2%	0.31	0.59	0.73
DUnet	**46.9%**	**0.41**	0.61	**0.8**

Noted that the obtained highest value is displayed in bold.

**Table 2:** Performance of models with different network architectures

	SR-PRE			SR-DCC		
Validation set	SC6K	COACH420	BU48	SC6K	COACH420	BU48
ResNet	40.2%	31.1%	39.3%	48.9%	46.9%	51.6%
ResNet + SA	45.5%	33.2%	40.1%	49.3%	46.9%	56.4%
DenseNet	45.6%	32.5%	**48.3%**	49.2%	45%	**66.1%**
DenseNet + SA (DUnet)	**48.4%**	**35.5%**	43.6%	**52%**	**47.6%**	58.1%

Noted that all networks were basically integrated by DUnet. Self-attention: SR-PRE; SR while precision ≥ 50%; SR-DCC: SR while predicted and real pocket DCC ≤ 4 Å.

### Assessing the predicted binding sites affected enzyme catalysis

To assess the capability of DUnet for predicting of the binding sites that can critically affect enzyme catalysis, we conducted a case study by predicting the substrate binding sites of KvAP and BaP4H using PUResNet, PointSite, and DUnet. Mutating residue to Ala resulting in less than 20% activity alteration was considered to be weakly bound residues that occasionally bind to the substrate [36]. Residues involved in electron transport during catalysis can lead to more than 40% activity loss via Ala mutation and were considered significant substrate binding or catalytic residues [37, 38]. In this study, we conducted Ala scan for the predicted binding sites to show their significance to enzyme catalysis.

**Figure 3:**
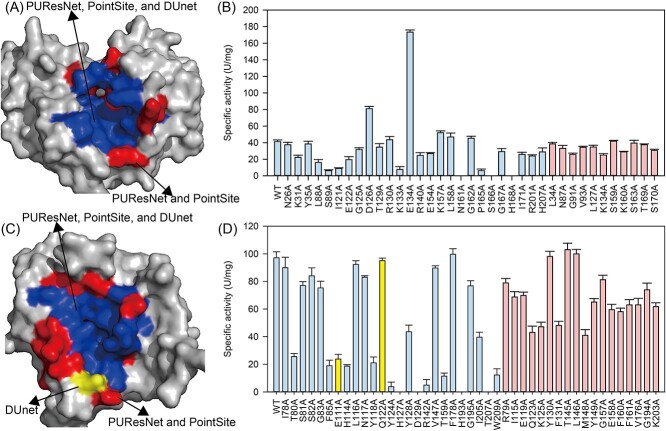
Validation of the predicted binding sites affected enzyme activity. The predicted binding sites of KvAP (A) and BaP4H (C), the predicted binding sites using PUResNet, PointSite, and DUnet were indicated in figure (detailed information provided in Tables S2 and S3). The specific activity changes while the predicted binding sites mutated to Ala based on KvAP (B) and BaP4H (D).

The structure of KvAP was modeled by AlphaFold-2, and PUResNet, PointSite, and DUnet predicted 40, 36, and 26 substrate binding sites (Table S2). The binding sites predicted by DUnet was fully covered by PUResNet and PointSite (Fig. 3A). All of the predicted binding sites were mutated to Ala and recombinantly expressed in *Escherichia coli*. The purified enzyme was used for measuring its activity against nitrophenyl phosphate (p-NPP) (Fig. S1). The results suggested that 35.9%, 41.2%, and 53.8% of the predicted substrate binding site by PUResNet, PointSite, and DUnet on KvAP resulted in more than 40% activity alterations (Fig. 3B). However, all of the predicted binding sites other than DUnet predicted ones showed less than 40% activity change upon mutating to Ala.

The crystallized structure of BaP4H (PDB: 5v7y) was used [31], and PUResNet, PointSite, and DUnet predicted 43, 41, and 25 substrate binding sites (Fig. 3C). The binding sites of E111 and Q122 were predicted only by DUnet (Table S3). The variants BaP4H were expressed and purified from *E. coli* (Fig. S2) and measured their activity against peptide (GPP)_5_. Ala scan results showed that 42.5%, 43.6%, and 56% of the predicted substrate binding site by PUResNet, PointSite, and DUnet on BaP4H resulted in more than 40% activity alterations (Fig. 3D). Moreover, 83.3% of the predicted sites other than DUnet predicted ones with less than 40% activity changes upon mutagenesis. These results suggested that DUnet-predicted binding sites covered more than 50% of the significant catalytic residues [39]. Additionally, predicting the binding sites in close proximity to catalytic center is suggested to critically affect enzyme activity, and these sites are important target sites for enzyme engineering [40].

### Evaluating the predicted binding sites affected virtual mutagenesis

**Figure 4:**
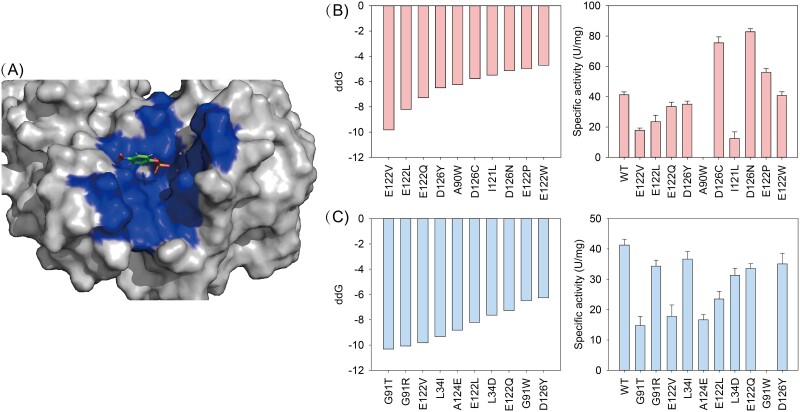
Validation of virtual mutagenesis. (A) Docking p-NPP into KvAP using Rosetta dock. The common predicted binding sites by PUResNet, PointSite, and DUnet are shown. The top-ranked 10 ddG achieved by virtual mutagenesis based on the predicted binding sites by DUnet (B) and PUResNet (C), and the correlated activity changes upon single mutation based on DUnet (B) and PUResNet (C) results.

Due to the predicted binding sites of KvAP using PUResNet covered PointSite and DUnet, we prepared two list containing candidate residues for virtual mutagenesis (Table S2). The binding pose of KvAP versus p-NPP was achieved by Rosetta dock [41] (Fig. S3), and the docking pose with the lowest binding score within a certain contact surface (dG_cross/SASA) was selected for virtual saturation mutagenesis [42] (Fig. 4A). The top-ranked 10 single mutations that can promote enzyme-substrate binding varied while the predicted binding sites were different, and four single mutations existed in the list from both PUResNet and DUnet. These variants were expressed and purified from *E. coli* and measured their activity against p-NPP.

As shown in Figure 4B, based on the list of DUnet, three variants improved the specific activity of KvAP, namely, D126N, which promoted the highest activity by 1-fold, and E122P and D126C, which promoted the activity by 35.6% and 82.9%. In comparison, none of the top-ranked 10 single mutations converted higher KvAP activity (Fig. 4C). Although PUResNet and PointSite accurately predicted three positive variants as the binding sites, the identification of additional binding sites altered the top-ranked single mutations. This necessitated further experimental work to achieve the positive results. Moreover, the predicted binding sites that are far from the real binding sites showed minor contribution to enzyme activity engineering.

### The combined tool for substrate binding sites prediction and molecular docking

**Figure 5:**
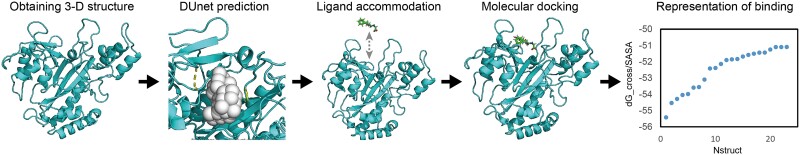
The protocol of DUnet-assisted molecular docking. The 3D structure of target enzyme can be obtained from PDB or AlphaFold database, followed by binding sites prediction using DUnet. The obtained center of the binding sites is used for accommodating substrate by Gromacs Editconf module and combined with structure file for molecular docking using Rosetta dock. The resulted binding score can be used to evaluate the potential interaction between novel enzymes and desired substrate.

Identification of substrate binding residues combined with the docking method can assist the discovery of functional enzymes [26, 43]. For the case study, we introduced the combined tools by predicting substrate binding sites of target enzyme followed by performing molecular docking. The advantage of this tool is to quickly annotate the binding sites while many structures are available for molecular docking. By using this tool, the center of the predicted binding sites was exported for accommodating of ligand using the Gromacs Editconf module. Then, molecular docking was performed by Rosetta dock for calculating the binding score (Fig. 5).

In addition, to enable convenient use of DUnet for the downstream process, the AlphaFold-2 modeled structures (accessed on 1 October 2023) containing 542 380 protein data entries (structures less than 100 kb were removed) annotated by Swiss-Prot were predicted using DUnet [32]. The predicted results, including ligand binding sites and the exact binding residues within proteins, have been deposited in GitHub for public access. These results may provide support for protein/enzyme engineering and help in the progress of enzyme activity design protocols.

## Discussion

This study used structural information to train a network for the prediction of substrate binding sites and developed a DUnet based on DenseNet, UNet, and SA [44] to perform deep feature extraction [19–21]. DUnet trained using enlarged training set demonstrating better model accuracy. DUnet was employed to predict the substrate binding sites, showing SRs of 48.4%, 35.5%, and 43.6% at a precision ≥50%, and 52%, 47.6%, and 58.1% while the distance of predicted and real center is ≤4 Å based on SC6K, COACH420, and BU48 validation datasets. The predicted binding sites of KvAP and BaP4H using PUResNet, PointSite, and DUnet were assessed, showing that DUnet exhibited the highest capability for predicting the key sites that affect enzyme catalysis. In addition, the predicted binding sites of KvAP were used for virtual mutagenesis, suggesting that correctly predicting the key binding sites can ultimately benefit the SR of virtual mutagenesis. DUnet was applied to predict the binding sites of Swiss-Prot annotated proteins, and the downstream protocol by assisting molecular docking was shown.

Dataset and network architectures can affect model accuracy [45]. Unlike sequential-based models [46], the 3D CNN-based model used in this study simply relied on structural information to predict substrate binding sites. The interactions between proteins and ligands can be learned from structures [47], but were not provided from sequences highlighting the significance for building structural-based models [48]. Meanwhile, we showed data augmentation can further promote model accuracy [20, 49]. The utility of DenseNet in solving protein-based tasks highlighted its performance in extracting features of 3D targets [26], with DenseNet presenting better performance during benchmark tests than ResNet [25]. To address the limitation of CNN for extracting features from a partial region of the object [29], self-attention was introduced for building the network of DUnet and finally achieved better performance.

The significant contribution of DUnet is to narrow the prediction range of key binding sites, thereby eliminating many fake “hot spots.” Selecting key pocket residues is important for enzyme activity and selectivity engineering [12, 13, 50]. Few residues actually involved in enzyme-induced catalysis despite the presence of many residues within 6 Å of the substrate [51]. The residues within the substrate consist of those residues for providing nonbound interactions to support substrate orientation [52] and are usually desired for modification [53]. Residues involved in electron transfer or nucleophilic attack are critical for the catalysis, leading to dramatic activity loss upon mutagenesis [54]. Through a comparison of predicted binding sites by DUnet and the other DL-based tools evaluated in this study, we showed that the region predicted by DUnet was smaller and was supported by experiments based on KvAP and BaP4H. These results indicated that DUnet efficiently predicted pocket residues for enzyme engineering.

Here, we introduced DUnet for predicting the protein–ligand binding sites by capturing the structural features and contributing to reducing the range of key pocket sites. The model was optimized by data augmentation and rebuilding the network. However, although DUnet could assist in enzyme activity engineering, the SR of DUnet for correctly predicting the real pocket location (DCC) was still limited by 47.6%–58.1% and must be further improved. Therefore, future research should focus on continuous enlargement of the dataset by manually curating data from public datasets and using more sophisticated networks.

## Methods

### Data preparation

The protein structures were converted into a 3D grid and represented as voxels with a size of 2 Å^3^ (Fig. S4). Protein feature extraction was conducted based on physical atomic characteristics, with each atom described by 18 atomic features, such as atom types, hybridization, in bound with other heavy atoms or heteroatoms, and partial charge. [55]. The output array was in the shape of 36 × 36 × 36 × 1, with the atomic features replaced with values of 1 and 0 to indicate the ligand binding or nonbinding. The ligand binding sites were extracted as array with an initial shape of 36 × 36 × 36 × 18.

The protein data in the scPDB_5020 dataset were cleaned to remove the water molecules, metals, and ligands. The scPDB5020_r dataset was prepared by rotating the protein and cavity structures in scPDB_5020 by 15° [56] (Fig. S5). SC6K, COACH420, and BU48 were used as validation sets [20]. The curated CAMEO dataset by Xu *et al*. was used as a blind test set [34, 35].

### Model architecture

DUnet was derived from DenseNet [26], SA [29], and UNet, containing an encoder side for extracting features and a decoder side for recovering sample size (Fig. 1). DenseNet was integrated in the decoder side by connecting the layers from both encoder and decoder sides. The representation of DenseNet as below, where *x*_0_, *x*_1_, *x*_(l − 1)_ are the features of the first, second, and (*l*–1)th layers, and *D* is the combination of convolution.

DenseNet(*l*) *= D_l_*([*x*_0_, *x*_1_, *…, x*_(*l*–1)_])*.*

The SA block used in this study by combining features from two separate layers, and the output of SA was used to connect with the convolution layers. The representation of SA as below, where *Q*, *K*, and *V* are vectors of queries, keys, and values of dimension *d_k_*, where *d_k_* is the size of the attention keys.


$$ \mathrm{Attention}\left(Q,K,V\right)=\mathrm{softmax}\left(\frac{QKT}{\sqrt{dk}}\right)V $$

In addition, we used BatchNorm3D after convolution to reduce internal covariate shift, and MaxPool3D to reduce the spatial dimensions of the input while retaining the most salient features. The sigmoid activation function was used in the decoder side to finally convert the point value to 0 and 1 to match the labels.

### Model evaluation

DCC was used to measure the barycenter distance between the predicted binding sites and actual ligand. The evaluation metrics including precision, sensitivity, and specificity were used based on the TP, TN, FP, and FN. The TP, TN, FP, and FN were based on the true or false of the predicted binding sites.

### Architecture of Rosetta script

The Rosetta script for molecular docking was developed using HighResDocker mover, InterfaceAnalyzerMover [57], MinMover, and FastRelax [58] mover. The substrate binding pocket was achieved by DUnet prediction, and the ligand was accommodated to the substrate binding pocket using Gromacs-2020 Editconf module (Uppsala University, Uppsala, Sweden) [56]. The ligand file was processed using Multiwfn [59] and Sobtop (Tian Lu, Sobtop, Version 1.0, http://sobereva.com/soft/Sobtop), combined with the enzyme structural file, and then advanced to molecular docking (the script was provided within the GitHub repository).

### Strains and plasmids

The plasmid pET-22b (+) and *E. coli* BL21 (DE3) were used for expressing KvAP (UniProt entry: A0A0B7G7J5), BaP4H [31], and related variants (Table S5).

### Plasmid construction

The genes encoding AP and P4H were synthesized and cloned into pET-22b via *Nde*I and *Blp*I sites to obtain the plasmids pET-22b/*AP* and pET-22b/*P4H*, respectively (GenScript). The plasmids encoding the variants carrying single residue mutations were constructed by PCR using pET-22b/*KvAP* or pET-22b/*BaP4H* as template and corresponding primers are listed in Tables S7 and S8. The PCR products were purified and circularized using the Blunting Kination Ligation Kit (TaKaRa, Dalian, China).

### Protein expression and purification

The plasmids encoding KvAP and BaP4H variants were transformed into *E. coli*, and the colony was inoculated into Luria–Bertani (LB) medium supplemented with 50 μg/ml ampicillin to cultivate at 37°C for 10 h. The seed culture was transferred into Terrific broth supplemented with 50 μg/ml ampicillin and cultivated until the cell density (OD_600_) reached 1.0. Protein induction was carried out by supplementing 0.1 mM isopropylthio-β-galactoside (IPTG), and the cells were continuously cultivated under 25°C for 30 h. The cells were harvested by centrifugation and resuspended in Tris-HCl (50 mM, pH 8.0) for ultra-sonification. The obtained solution was centrifuged at 10 000 × g and the supernatant was subjected to affinity chromatography using His-Trap column (GE Healthcare, New York, USA) and size-exclusion chromatography using Superdex 75 column (GE Healthcare, USA). The protein concentration was determined using a Bradford Protein Assay Kit (Beyotime, Shanghai, China), and SDS-PAGE was conducted using a 12% Tris-glycine gel (Thermo Fisher Scientific, Shanghai, China).

### Acid phosphatase (AP) activity assay

The specific activity of AP against p-NPP was measured accordingly [60]. The substrate solution comprised 200 mM p-NPP disodium salt and 50 mM acetate buffer (pH 5.0). During the reaction, 10 μl of sample protein were added to 200 μl of substrate solution and prewarmed at 37°C for 5 min. Then, the mixture was incubated at 37°C for 20 min and the reaction was terminated by adding 1 ml of 0.5 M NaOH. The produced p-NP was measured at 405 nm. One unit of AP activity was defined as 1 μmol p-NP produced per minute.

### P4H activity assay

The substrate solution for the P4H assay consisted of 5 mM (GPP)_5_, 10 mM α-ketoglutarate, 0.5 mM FeSO_4_, 1.5 mM l-ascorbate, 0.5% (v/v) DMSO, and 50 mM Tris buffer (pH 6.5). During the reaction, 20 μl of the sample protein were added to 180 μl of substrate solution and incubated at 30°C for 1 h. The reaction was terminated by adding 50 μl of 6 M HCl. The produced free hydroxyproline (HXY) was detected using a Hydroxyproline Content Assay Kit (Sangon, Shanghai, China). One unit of P4H activity was defined as 1 μmol HXY produced per minute.

**Key Points**

- A 3D structure based network for protein or enzyme binding sites prediction was developed, showing 47.6%–58.1% accuracy for correctly locating the center of the binding pocket.
- The predicted key binding sites were validated based on acid phosphatase and proline 4-hydroxylase showing 54%–56% of the predicted sites was important for catalysis.
- The network was used to guide the modification of acid phosphatase, showing a new perspective for enzyme activity engineering.
- Predicting enzyme–substrate binding sites can assist the molecular docking protocol that may apply to novel enzyme discovery.

## Supplementary Material

Suplementary_material_bbae381

## Data Availability

The data supporting the findings of this study are available within the article and supplementary information. Other data and reagents are available from the corresponding authors upon reasonable request. Source data are provided with this paper. All the codes and datasets used this work are publicly available at: https://github.com/wangxinglong1990/DUnet.
