# Supplementary material for: Machine learning-assisted substrate binding pocket engineering based on structural information
Source: Brief Bioinform. 2024 Aug 5;25(5):bbae381. doi: 10.1093/bib/bbae381 (PMC11299021; doi:10.1093/bib/bbae381)
Supplement: Suplementary_material_bbae381 [file suplementary_material_bbae381.docx]

**Machine learning assisted substrate binding pocket engineering based on structural information**

Xinglong Wang^1,2,3^, Kangjie Xu^3^, Xuan Zeng^5^, Kai Linghu^3^, Beichen Zhao^3^, Shangyang Yu^3^, Kun Wang^3^, Shuyao Yu^3^, Xinyi Zhao^3^, Weizhu Zeng^3^, Kai Wang^5^, Jingwen Zhou^2,3,4,*^

^1^ School of food science and technology, Jiangnan University, 1800 Lihu Road, Wuxi, Jiangsu 214122, China;

^2^ Engineering Research Center of Ministry of Education on Food Synthetic Biotechnology and School of Biotechnology, Jiangnan University, 1800 Lihu Road, Wuxi, Jiangsu 214122, China;

^3^ Science Center for Future Foods, Jiangnan University, 1800 Lihu Road, Wuxi, Jiangsu 214122, China;

^4^ Jiangsu Province Engineering Research Center of Food Synthetic Biotechnology, Jiangnan University, Wuxi 214122, China.

^5^ Key Laboratory of Advanced Process Control for Light Industry (Ministry of Education), School of Internet of Things Engineering, Jiangnan University, 1800 Lihu Road, Wuxi, Jiangsu 214122, China.

* Corresponding author: Jingwen Zhou.

Science Center for Future Foods, Jiangnan University, 1800 Lihu Road, Wuxi, Jiangsu 214122, China.

Phone: +86-510-85914371, Fax: +86-510-85914371.

E-mail: zhoujw1982@jiangnan.edu.cn.

## Supplementary Figures


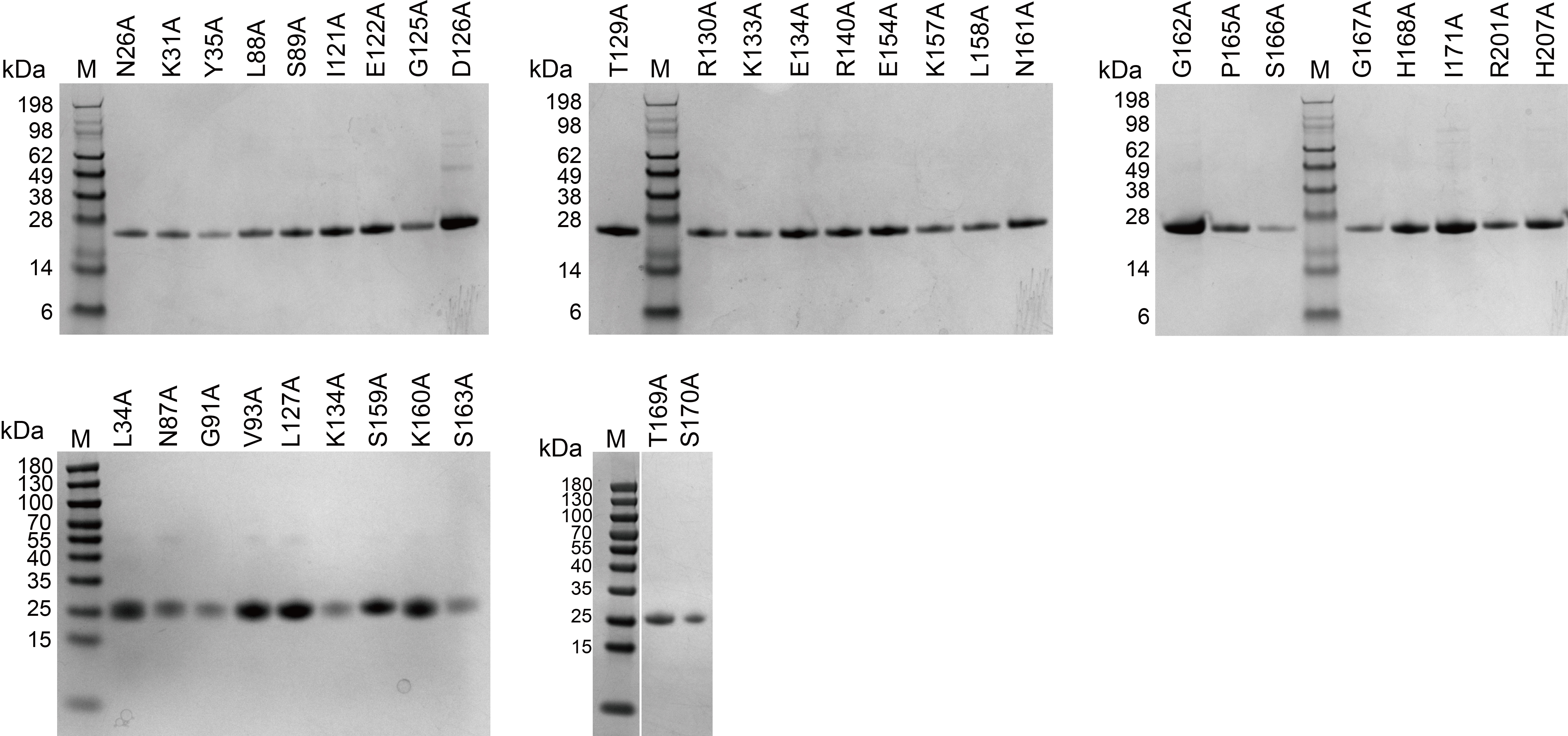


## Figure S1 Visualization of purified KvAP on SDS-PAGE

The KvAP variants were recombinantly expressed into *E. coli*. These variants were purified from the supernatant of intracellular fraction after fermentation.


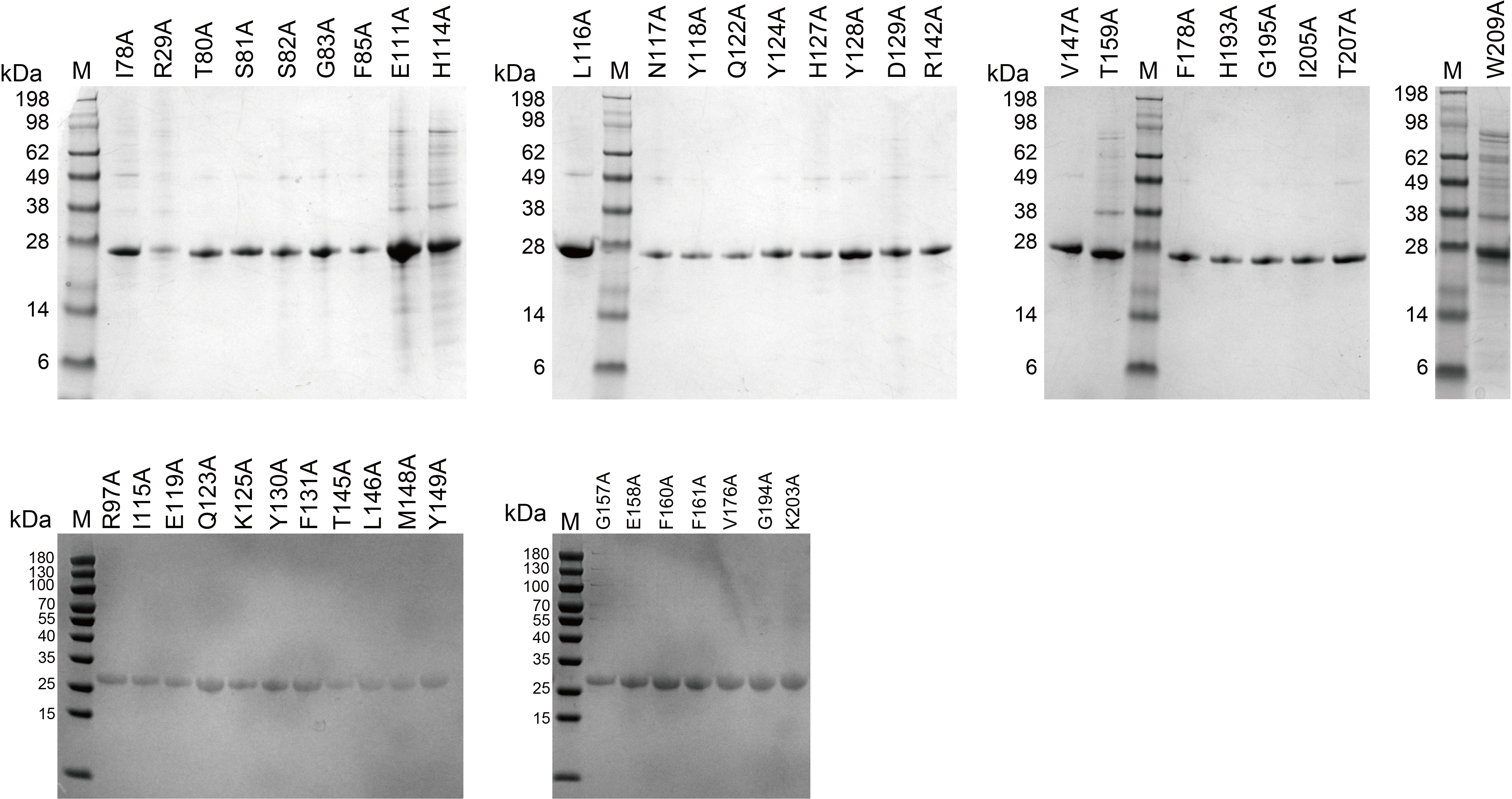


## Figure S2 Visualization of purified BaP4H variants on SDS-PAGE

The BaP4H variants were recombinantly expressed into *E. coli*. These variants were purified from the supernatant of intracellular fraction after fermentation.


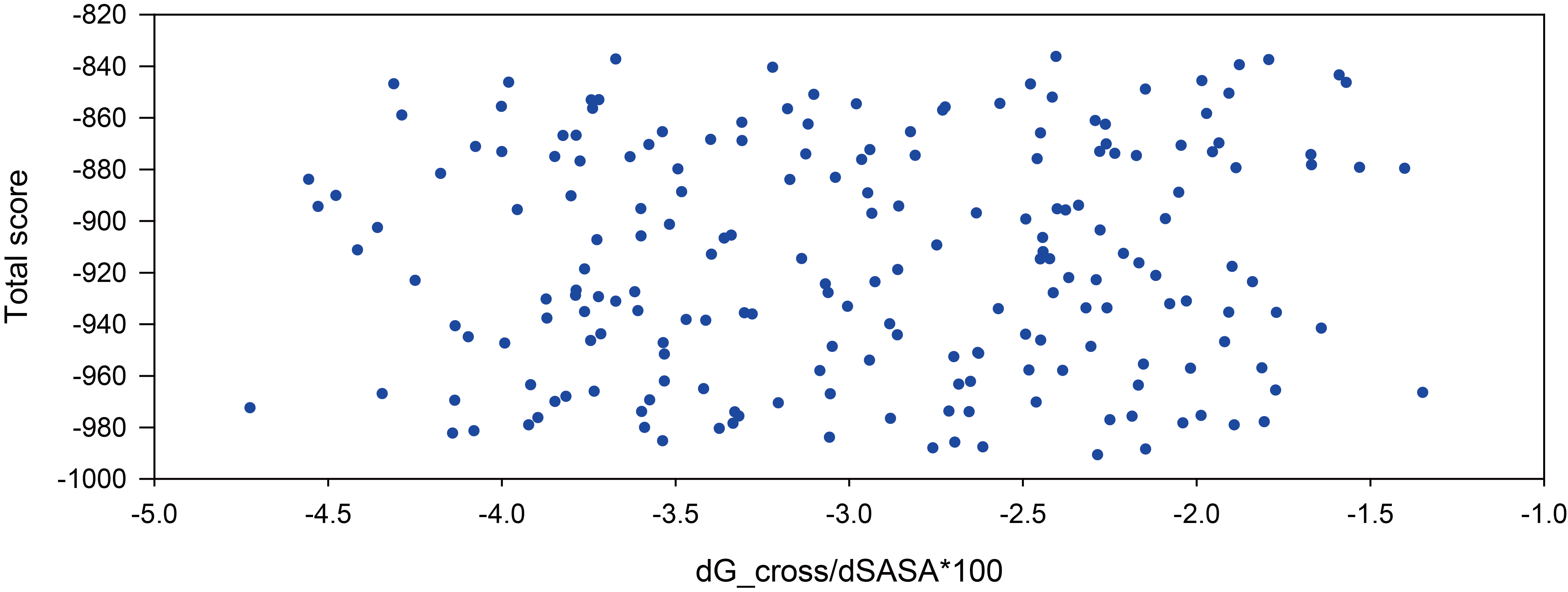


## Figure S3 Molecular docking of p-NPP against KvAP

Molecular docking was performed using Rosetta script [1], the obtained total_score and dG_cross (representing interface binding energy) dived SASA (solvent accessibility) of the corresponding docking complex were shown.

**
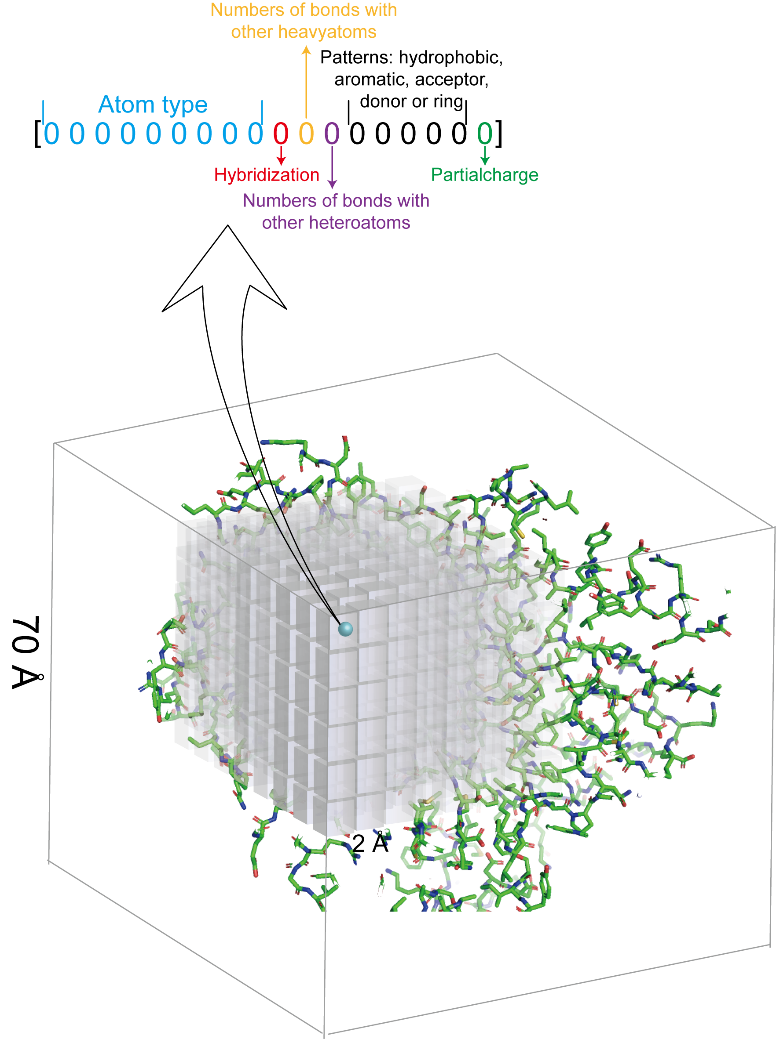
**

## Figure S4 Data representation

Protein structure was treated as 3-dimentional image at a size of 70 Å^3^. The image was represented as voxels at a size of 2 Å^3^ to ensure each voxel can only contain one atom. Atomic features were described according to the physical characters.

**
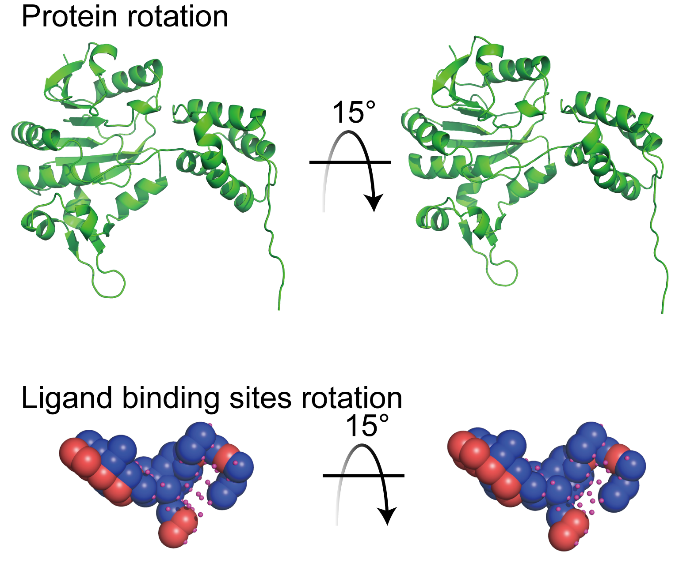
**

## Figure S5 Protein rotation

Rotation of protein and ligand binding sites in scPDB_5020 dataset using Gromacs-2020 by the editconf tool [2].

## Supplementary Tables

## Table S1 Performance of model trained on regular and enlarged dataset

|  | **Percentage of 𝐷𝐶𝐶 ≤ 4Å** | **DVO (𝐷𝐶𝐶 ≤ 4Å)** |
| --- | --- | --- |
| Trained with regular dataset | 43.3% | 0.64 |
| Trained with enlarged dataset | 45% | 0.78 |

We established a DenseNet and UNet-based network, and trained using scPDB_5020. In addition, the samples within scPDB_5020 was rotated by 15 degrees to create scPDB_5020_r training set. The network was then trained by combining samples from scPDB_5020 and scPDB_5020_r (The trained network was provided in our repository under the folder name of DenseNet). The success rate was evaluated by samples with distance of predicted and real center (DCC) ≤ 4Å, and the discretized volume overlap (DVO) when DCC ≤ 4Å. The DVO calculated by measuring the volumetric intersection between the predicted and actual binding site to their union. The obtained values shown in table was the average value of the 5-fold cross validation.

## Table S2 Exact predicted binding sites of KvAP using DL-methods.

|  | PUResNet | PointSite | DUnet |
| --- | --- | --- | --- |
| Sites | 26 | 26 | 26 |
|  | 31 | 31 | 31 |
|  | 34 | 34 |  |
|  | 35 | 35 | 35 |
|  | 87 |  |  |
|  | 88 | 88 | 88 |
|  | 89 | 89 | 89 |
|  | 90 | 90 | 90 |
|  | 91 | 91 |  |
|  | 93 | 93 |  |
|  | 121 | 121 | 121 |
|  | 122 | 122 | 122 |
|  | 124 | 124 |  |
|  | 125 | 125 | 125 |
|  | 126 | 126 | 126 |
|  | 127 | 127 |  |
|  | 128 | 128 |  |
|  | 129 | 129 | 129 |
|  | 130 | 130 | 130 |
|  | 133 | 133 | 133 |
|  | 134 |  |  |
|  | 140 | 140 | 140 |
|  | 154 | 154 | 154 |
|  | 157 | 157 | 157 |
|  | 158 | 158 | 158 |
|  | 159 |  |  |
|  | 160 |  |  |
|  | 161 | 161 | 161 |
|  | 162 | 162 | 162 |
|  | 163 | 163 |  |
|  | 165 | 165 | 165 |
|  | 166 | 166 | 166 |
|  | 167 | 167 | 167 |
|  | 168 | 168 | 168 |
|  | 169 | 169 |  |
|  | 170 | 170 |  |
|  | 171 | 171 | 171 |
|  | 201 | 201 | 201 |
|  | 206 | 206 |  |
|  | 207 | 207 | 207 |

## Table S3 Exact predicted binding sites of BaP4H using DL-methods.

|  | PUResNet | PointSite | DUnet |
| --- | --- | --- | --- |
| Sites | 78 | 78 | 78 |
|  | 79 | 79 |  |
|  | 80 | 80 | 80 |
|  | 81 | 81 | 81 |
|  | 82 | 82 | 82 |
|  | 83 | 83 | 83 |
|  | 85 | 85 | 85 |
|  |  |  | 111 |
|  | 114 | 114 | 114 |
|  | 115 | 115 |  |
|  | 116 | 116 | 116 |
|  | 117 |  | 117 |
|  | 118 | 118 | 118 |
|  | 119 | 119 |  |
|  |  |  | 122 |
|  | 123 | 123 |  |
|  | 124 | 124 | 124 |
|  | 125 | 125 |  |
|  | 126 |  |  |
|  | 127 | 127 | 127 |
|  | 128 | 128 | 128 |
|  | 129 | 129 | 129 |
|  | 130 | 130 |  |
|  | 131 | 131 |  |
|  | 142 | 142 | 142 |
|  | 145 | 145 |  |
|  | 146 | 146 |  |
|  | 147 | 147 | 147 |
|  | 148 |  |  |
|  | 149 | 149 |  |
|  | 157 | 157 |  |
|  | 158 | 158 |  |
|  | 159 | 159 | 159 |
|  | 160 | 160 |  |
|  | 161 | 161 |  |
|  | 176 | 176 |  |
|  | 178 | 178 | 178 |
|  | 193 | 193 | 193 |
|  | 194 | 194 |  |
|  | 195 | 195 | 195 |
|  | 196 | 196 |  |
|  | 203 | 203 |  |
|  | 205 | 205 | 205 |
|  | 206 | 206 |  |
|  | 207 | 207 | 207 |
|  | 209 | 209 | 209 |

## Table S4 Synthetic genes for KvAP and BaP4H.

| Enzyme name | Gene sequences (5′-3′) |
| --- | --- |
| KvAP | **CATATG**GCTCTAGTACCCCCAGGAAATGATGTTACGACCAAACCGGATCTTTATTACCTGACCAATGCTCAAGCGATTGACAGCCTGGCTTTGCTTCCGCCACCGCCTGCGGTTGGTTCGATTGCATTCCTGAACGATCAGGCAATGTATGAGCAAGGTCGGCTGTTACGCAACACCGAGCGCGGTAAATTGGCGGCAGAGGACGCGAACCTGTCTGCGGGTGGTGTCGCGAACGCGTTCAGCAGCGCGTTCGGCTCCCCGATTACCGAGAAGGACGCTCCGCAGCTGCACAAATTGCTGACCAACATGATTGAGGACGCAGGCGATCTCGCGACCCGTGGCGCAAAAGAAAAGTACATGCGCATCCGTCCGTTTGCGTTTTATGGCGTGAGCACCTGTAATACGACGGAACAAGACAAACTGAGCAAAAACGGTTCCTACCCGAGCGGTCATACTTCAATCGGCTGGGCAACTGCCTTGGTTCTGGCGGAGATCAACCCGCAACGTCAGAATGAAATCCTGAAACGTGGCTACGAACTGGGTGAAAGCCGTGTGATCTGCGGTTATCATTGGCAGTCCGATGTTGATGCCGCCCGTATCGTGGGCTCTGCGGTGGTTGCTACCCTGCACACCAATCCGGCGTTCCAGCAACAACTGCAGAAGGCTAAGGACGAATTTGCCAAGACCCAGAAGCACCACCACCACCACCAC**TGA**GATCCGGCTGCTAACAAAGCCCGAAAGGAAGCTGAGTTGGCTGCTGCCACC**GCTGAGC** |
| BaP4H | **CATATG**GCGACGAACAACAACCAAATTGGCGAAAATAAGGAACAAACTATCTTCGATCATAAAGGCAACGTTATCAAAACTGAGGACCGTGAAATCCAGATCATTTCTAAGTTTGAAGAGCCGCTGATTGTGGTGCTGGGCAACGTGCTGAGCGATGAGGAGTGCGATGAACTGATTGAGCTGTCTAAGTCCAAACTGGCTCGTTCTAAAGTGGGTTCTAGCCGTGATGTGAACGACATTCGTACTAGCTCTGGTGCCTTCCTGGACGATAACGAACTGACGGCTAAAATCGAAAAACGCATCTCCTCCATCATGAATGTTCCGGCCTCTCACGGTGAAGGTCTGCACATCCTGAACTACGAAGTTGATCAGCAGTACAAGGCACATTACGACTACTTCGCAGAACACTCCCGTAGCGCAGCAAACAACCGTATTTCCACCCTGGTCATGTATCTGAATGACGTGGAAGAAGGTGGTGAGACCTTCTTTCCAAAGCTGAATCTGTCCGTTCACCCACGTAAAGGTATGGCAGTCTACTTCGAATATTTCTACCAGGACCAGTCTCTGAACGAACTGACCCTGCATGGTGGTGCGCCAGTTACCAAAGGCGAGAAATGGATCGCGACTCAGTGGGTGCGTCGCGGTACTTATAAGCCTCCTGGCCCACCAGGTCCGCCGGGCCACCACCACCACCACCAC**TGA**GATCCGGCTGCTAACAAAGCCCGAAAGGAAGCTGAGTTGGCTGCTGCCACCGCTGAGCAATAACTAGCATAACC**CCTTGG** |

The restriction site of *Nde*I, *Blp*I and *StyI* were underlined. The start and end codon were shown in red and green. The none-encoding region after end codon was wave lined.

## Table S5 Primers used to conduct single mutations for BaP4H variants.

| Primers | Sequences (5′-3′) | Remarks |
| --- | --- | --- |
| B1F | GCACGTACTAGCTCTGGTGCCTTCCTGGACGATAACGAACTGACG | Forward primer for BaP4H I78A mutation |
| B1R | GTCGTTCACATCACGGCTAGAACCCACTTTAGAACGAGCCAGTTT | Reverse primer for BaP4H I78A mutation |
| B2F | GCAAGCTCTGGTGCCTTCCTGGACGATAACGAACTGACGGCTAAA | Forward primer for BaP4H T80A mutation |
| B2R | ACGAATGTCGTTCACATCACGGCTAGAACCCACTTTAGAACGAGC | Reverse primer for BaP4H T80A mutation |
| B3F | GCATCTGGTGCCTTCCTGGACGATAACGAACTGACGGCTAAAATC | Forward primer for BaP4H S81A mutation |
| B3R | AGTACGAATGTCGTTCACATCACGGCTAGAACCCACTTTAGAACG | Reverse primer for BaP4H S81A mutation |
| B4F | GCAGGTGCCTTCCTGGACGATAACGAACTGACGGCTAAAATCGAA | Forward primer for BaP4H S82A mutation |
| B4R | GCTAGTACGAATGTCGTTCACATCACGGCTAGAACCCACTTTAGA | Reverse primer for BaP4H S82A mutation |
| B5F | GCAGCCTTCCTGGACGATAACGAACTGACGGCTAAAATCGAAAAA | Forward primer for BaP4H G83A mutation |
| B5R | AGAGCTAGTACGAATGTCGTTCACATCACGGCTAGAACCCACTTT | Reverse primer for BaP4H G83A mutation |
| B6F | GCACTGGACGATAACGAACTGACGGCTAAAATCGAAAAACGCATC | Forward primer for BaP4H F85A mutation |
| B6R | GGCACCAGAGCTAGTACGAATGTCGTTCACATCACGGCTAGAACC | Reverse primer for BaP4H F85A mutation |
| B7F | GCAGGTCTGCACATCCTGAACTACGAAGTTGATCAGCAGTACAAG | Forward primer for BaP4H E111A mutation |
| B7R | ACCGTGAGAGGCCGGAACATTCATGATGGAGGAGATGCGTTTTTC | Reverse primer for BaP4H E111A mutation |
| B8F | GCAATCCTGAACTACGAAGTTGATCAGCAGTACAAGGCACATTAC | Forward primer for BaP4H H114A mutation |
| B8R | CAGACCTTCACCGTGAGAGGCCGGAACATTCATGATGGAGGAGAT | Reverse primer for BaP4H H114A mutation |
| B9F | GCAAACTACGAAGTTGATCAGCAGTACAAGGCACATTACGACTAC | Forward primer for BaP4H L116A mutation |
| B9R | GATGTGCAGACCTTCACCGTGAGAGGCCGGAACATTCATGATGGA | Reverse primer for BaP4H L116A mutation |
| B10F | GCATACGAAGTTGATCAGCAGTACAAGGCACATTACGACTACTTC | Forward primer for BaP4H N117A mutation |
| B10R | CAGGATGTGCAGACCTTCACCGTGAGAGGCCGGAACATTCATGAT | Reverse primer for BaP4H N117A mutation |
| B11F | GCAGAAGTTGATCAGCAGTACAAGGCACATTACGACTACTTCGCA | Forward primer for BaP4H Y118A mutation |
| B11R | GTTCAGGATGTGCAGACCTTCACCGTGAGAGGCCGGAACATTCAT | Reverse primer for BaP4H Y118A mutation |
| B12F | GCACAGTACAAGGCACATTACGACTACTTCGCAGAACACTCCCGT | Forward primer for BaP4H Q122A mutation |
| B12R | ATCAACTTCGTAGTTCAGGATGTGCAGACCTTCACCGTGAGAGGC | Reverse primer for BaP4H Q122A mutation |
| B13F | GCAAAGGCACATTACGACTACTTCGCAGAACACTCCCGTAGCGCA | Forward primer for BaP4H Y124A mutation |
| B13R | CTGCTGATCAACTTCGTAGTTCAGGATGTGCAGACCTTCACCGTG | Reverse primer for BaP4H Y124A mutation |
| B14F | GCATACGACTACTTCGCAGAACACTCCCGTAGCGCAGCAAACAAC | Forward primer for BaP4H H127A mutation |
| B14R | TGCCTTGTACTGCTGATCAACTTCGTAGTTCAGGATGTGCAGACC | Reverse primer for BaP4H H127A mutation |
| B15F | GCAGACTACTTCGCAGAACACTCCCGTAGCGCAGCAAACAACCGT | Forward primer for BaP4H Y128A mutation |
| B15R | ATGTGCCTTGTACTGCTGATCAACTTCGTAGTTCAGGATGTGCAG | Reverse primer for BaP4H Y128A mutation |
| B16F | GCATACTTCGCAGAACACTCCCGTAGCGCAGCAAACAACCGTATT | Forward primer for BaP4H D129A mutation |
| B16R | GTAATGTGCCTTGTACTGCTGATCAACTTCGTAGTTCAGGATGTG | Reverse primer for BaP4H D129A mutation |
| B17F | GCAATTTCCACCCTGGTCATGTATCTGAATGACGTGGAAGAAGGT | Forward primer for BaP4H R142A mutation |
| B17R | GTTGTTTGCTGCGCTACGGGAGTGTTCTGCGAAGTAGTCGTAATG | Reverse primer for BaP4H R142A mutation |
| B18F | GCAATGTATCTGAATGACGTGGAAGAAGGTGGTGAGACCTTCTTT | Forward primer for BaP4H V147A mutation |
| B18R | CAGGGTGGAAATACGGTTGTTTGCTGCGCTACGGGAGTGTTCTGC | Reverse primer for BaP4H V147A mutation |
| B19F | GCATTCTTTCCAAAGCTGAATCTGTCCGTTCACCCACGTAAAGGT | Forward primer for BaP4H T159A mutation |
| B19R | CTCACCACCTTCTTCCACGTCATTCAGATACATGACCAGGGTGGA | Reverse primer for BaP4H T159A mutation |
| B20F | GCAGAATATTTCTACCAGGACCAGTCTCTGAACGAACTGACCCTG | Forward primer for BaP4H F178A mutation |
| B20R | GTAGACTGCCATACCTTTACGTGGGTGAACGGACAGATTCAGCTT | Reverse primer for BaP4H F178A mutation |
| B21F | GCAGGTGGTGCGCCAGTTACCAAAGGCGAGAAATGGATCGCGACT | Forward primer for BaP4H H193A mutation |
| B21R | CAGGGTCAGTTCGTTCAGAGACTGGTCCTGGTAGAAATATTCGAA | Reverse primer for BaP4H H193A mutation |
| B22F | GCAGCGCCAGTTACCAAAGGCGAGAAATGGATCGCGACTCAGTGG | Forward primer for BaP4H G195A mutation |
| B22R | ACCATGCAGGGTCAGTTCGTTCAGAGACTGGTCCTGGTAGAAATA | Reverse primer for BaP4H G195A mutation |
| B23F | GCAGCGACTCAGTGGGTGCGTCGCGGTACTTATAAGCCTCCTGGC | Forward primer for BaP4H I205A mutation |
| B23R | CCATTTCTCGCCTTTGGTAACTGGCGCACCACCATGCAGGGTCAG | Reverse primer for BaP4H I205A mutation |
| B24F | GCACAGTGGGTGCGTCGCGGTACTTATAAGCCTCCTGGCCCACCA | Forward primer for BaP4H T207A mutation |
| B24R | CGCGATCCATTTCTCGCCTTTGGTAACTGGCGCACCACCATGCAG | Reverse primer for BaP4H T207A mutation |
| B25F | GCAGTGCGTCGCGGTACTTATAAGCCTCCTGGCCCACCAGGTCCG | Forward primer for BaP4H W209A mutation |
| B25R | CTGAGTCGCGATCCATTTCTCGCCTTTGGTAACTGGCGCACCACC | Reverse primer for BaP4H W209A mutation |
| B26F | GCAACTAGCTCTGGTGCCTTCCTGGACGATAACGAACTGACGGCT | Forward primer for BaP4H R79A mutation |
| B26R | AATGTCGTTCACATCACGGCTAGAACCCACTTTAGAACGAGCCAG | Reverse primer for BaP4H R79A mutation |
| B27F | GCACTGAACTACGAAGTTGATCAGCAGTACAAGGCACATTACGAC | Forward primer for BaP4H I115A mutation |
| B27R | GTGCAGACCTTCACCGTGAGAGGCCGGAACATTCATGATGGAGGA | Reverse primer for BaP4H I115A mutation |
| B28F | GCAGTTGATCAGCAGTACAAGGCACATTACGACTACTTCGCAGAA | Forward primer for BaP4H E119A mutation |
| B28R | GTAGTTCAGGATGTGCAGACCTTCACCGTGAGAGGCCGGAACATT | Reverse primer for BaP4H E119A mutation |
| B29F | GCATACAAGGCACATTACGACTACTTCGCAGAACACTCCCGTAGC | Forward primer for BaP4H Q123A mutation |
| B29R | CTGATCAACTTCGTAGTTCAGGATGTGCAGACCTTCACCGTGAGA | Reverse primer for BaP4H Q123A mutation |
| B30F | GCAGCACATTACGACTACTTCGCAGAACACTCCCGTAGCGCAGCA | Forward primer for BaP4H K125A mutation |
| B30R | GTACTGCTGATCAACTTCGTAGTTCAGGATGTGCAGACCTTCACC | Reverse primer for BaP4H K125A mutation |
| B31F | GCATTCGCAGAACACTCCCGTAGCGCAGCAAACAACCGTATTTCC | Forward primer for BaP4H Y130A mutation |
| B31R | GTCGTAATGTGCCTTGTACTGCTGATCAACTTCGTAGTTCAGGAT | Reverse primer for BaP4H Y130A mutation |
| B32F | GCACTGGTCATGTATCTGAATGACGTGGAAGAAGGTGGTGAGACC | Forward primer for BaP4H T145A mutation |
| B32R | GGAAATACGGTTGTTTGCTGCGCTACGGGAGTGTTCTGCGAATGC | Reverse primer for BaP4H T145A mutation |
| B33F | GCAGTCATGTATCTGAATGACGTGGAAGAAGGTGGTGAGACCTTC | Forward primer for BaP4H L146A mutation |
| B33R | GGTGGAAATACGGTTGTTTGCTGCGCTACGGGAGTGTTCTGCGAA | Reverse primer for BaP4H L146A mutation |
| B34F | GCATATCTGAATGACGTGGAAGAAGGTGGTGAGACCTTCTTTCCA | Forward primer for BaP4H M148A mutation |
| B34R | GACCAGGGTGGAAATACGGTTGTTTGCTGCGCTACGGGAGTGTTC | Reverse primer for BaP4H M148A mutation |
| B35F | GCACTGAATGACGTGGAAGAAGGTGGTGAGACCTTCTTTCCAAAG | Forward primer for BaP4H Y149A mutation |
| B35R | CATGACCAGGGTGGAAATACGGTTGTTTGCTGCGCTACGGGAGTG | Reverse primer for BaP4H Y149A mutation |
| B36F | GCAGAGACCTTCTTTCCAAAGCTGAATCTGTCCGTTCACCCACGT | Forward primer for BaP4H G157A mutation |
| B36R | ACCTTCTTCCACGTCATTCAGATACATGACCAGGGTGGAAATACG | Reverse primer for BaP4H G157A mutation |
| B37F | GCAACCTTCTTTCCAAAGCTGAATCTGTCCGTTCACCCACGTAAA | Forward primer for BaP4H E158A mutation |
| B37R | ACCACCTTCTTCCACGTCATTCAGATACATGACCAGGGTGGAAAT | Reverse primer for BaP4H E158A mutation |
| B38F | GCATTTCCAAAGCTGAATCTGTCCGTTCACCCACGTAAAGGTATG | Forward primer for BaP4H F160A mutation |
| B38R | GGTCTCACCACCTTCTTCCACGTCATTCAGATACATGACCAGGGT | Reverse primer for BaP4H F160A mutation |
| B39F | GCACCAAAGCTGAATCTGTCCGTTCACCCACGTAAAGGTATGGCA | Forward primer for BaP4H F161A mutation |
| B39R | GAAGGTCTCACCACCTTCTTCCACGTCATTCAGATACATGACCAG | Reverse primer for BaP4H F161A mutation |
| B40F | GCATACTTCGAATATTTCTACCAGGACCAGTCTCTGAACGAACTG | Forward primer for BaP4H V176A mutation |
| B40R | TGCCATACCTTTACGTGGGTGAACGGACAGATTCAGCTTTGGAAA | Reverse primer for BaP4H V176A mutation |
| B41F | GCAGGTGCGCCAGTTACCAAAGGCGAGAAATGGATCGCGACTCAG | Forward primer for BaP4H G194A mutation |
| B41R | ATGCAGGGTCAGTTCGTTCAGAGACTGGTCCTGGTAGAAATATTC | Reverse primer for BaP4H G194A mutation |
| B42F | GCATGGATCGCGACTCAGTGGGTGCGTCGCGGTACTTATAAGCCT | Forward primer for BaP4H K203A mutation |
| B42R | CTCGCCTTTGGTAACTGGCGCACCACCATGCAGGGTCAGTTCGTT | Reverse primer for BaP4H K203A mutation |

The nucleotides generating mutation sites were underlined.

## Table S6 Primers used to conduct single mutations for KvAP variants.

| Primers | Sequences (5′-3′) | Remarks |
| --- | --- | --- |
| K1F | GCAGATGTTACGACCAAACCGGATCTTTATTACCTGACCAATGCT | Forward primer for KvAP N26A mutation |
| K1R | ATTTCCTGGGGGTACTAGAGCCATATGTATATCTCCTTCTTAAAG | Reverse primer for KvAP N26A mutation |
| K2F | GCACCGGATCTTTATTACCTGACCAATGCTCAAGCGATTGACAGC | Forward primer for KvAP K31A mutation |
| K2R | GGTCGTAACATCATTTCCTGGGGGTACTAGAGCCATATGTATATC | Reverse primer for KvAP K31A mutation |
| K3F | GCATACCTGACCAATGCTCAAGCGATTGACAGCCTGGCTTTGCTT | Forward primer for KvAP Y35A mutation |
| K3R | AAGATCCGGTTTGGTCGTAACATCATTTCCTGGGGGTACTAGAGC | Reverse primer for KvAP Y35A mutation |
| K4F | GCATCTGCGGGTGGTGTCGCGAACGCGTTCAGCAGCGCGTTCGGC | Forward primer for KvAP L88A mutation |
| K4R | GTTCGCGTCCTCTGCCGCCAATTTACCGCGCTCGGTGTTGCGTAA | Reverse primer for KvAP L88A mutation |
| K5F | GCAGCGGGTGGTGTCGCGAACGCGTTCAGCAGCGCGTTCGGCTCC | Forward primer for KvAP S89A mutation |
| K5R | CAGGTTCGCGTCCTCTGCCGCCAATTTACCGCGCTCGGTGTTGCG | Reverse primer for KvAP S89A mutation |
| K6F | GCAGAGGACGCAGGCGATCTCGCGACCCGTGGCGCAAAAGAAAAG | Forward primer for KvAP I121A mutation |
| K6R | CATGTTGGTCAGCAATTTGTGCAGCTGCGGAGCGTCCTTCTCGGT | Reverse primer for KvAP I121A mutation |
| K7F | GCAGACGCAGGCGATCTCGCGACCCGTGGCGCAAAAGAAAAGTAC | Forward primer for KvAP E122A mutation |
| K7R | AATCATGTTGGTCAGCAATTTGTGCAGCTGCGGAGCGTCCTTCTC | Reverse primer for KvAP E122A mutation |
| K8F | GCAGATCTCGCGACCCGTGGCGCAAAAGAAAAGTACATGCGCATC | Forward primer for KvAP G125A mutation |
| K8R | TGCGTCCTCAATCATGTTGGTCAGCAATTTGTGCAGCTGCGGAGC | Reverse primer for KvAP G125A mutation |
| K9F | GCACTCGCGACCCGTGGCGCAAAAGAAAAGTACATGCGCATCCGT | Forward primer for KvAP D126A mutation |
| K9R | GCCTGCGTCCTCAATCATGTTGGTCAGCAATTTGTGCAGCTGCGG | Reverse primer for KvAP D126A mutation |
| K10F | GCACGTGGCGCAAAAGAAAAGTACATGCGCATCCGTCCGTTTGCG | Forward primer for KvAP T129A mutation |
| K10R | CGCGAGATCGCCTGCGTCCTCAATCATGTTGGTCAGCAATTTGTG | Reverse primer for KvAP T129A mutation |
| K11F | GCAGGCGCAAAAGAAAAGTACATGCGCATCCGTCCGTTTGCGTTT | Forward primer for KvAP R130A mutation |
| K11R | GGTCGCGAGATCGCCTGCGTCCTCAATCATGTTGGTCAGCAATTT | Reverse primer for KvAP R130A mutation |
| K12F | GCAGAAAAGTACATGCGCATCCGTCCGTTTGCGTTTTATGGCGTG | Forward primer for KvAP K133A mutation |
| K12R | TGCGCCACGGGTCGCGAGATCGCCTGCGTCCTCAATCATGTTGGT | Reverse primer for KvAP K133A mutation |
| K13F | GCAAAGTACATGCGCATCCGTCCGTTTGCGTTTTATGGCGTGAGC | Forward primer for KvAP E134A mutation |
| K13R | TTTTGCGCCACGGGTCGCGAGATCGCCTGCGTCCTCAATCATGTT | Reverse primer for KvAP E134A mutation |
| K14F | GCACCGTTTGCGTTTTATGGCGTGAGCACCTGTAATACGACGGAA | Forward primer for KvAP R140A mutation |
| K14R | GATGCGCATGTACTTTTCTTTTGCGCCACGGGTCGCGAGATCGCC | Reverse primer for KvAP R140A mutation |
| K15F | GCACAAGACAAACTGAGCAAAAACGGTTCCTACCCGAGCGGTCAT | Forward primer for KvAP E154A mutation |
| K15R | CGTCGTATTACAGGTGCTCACGCCATAAAACGCAAACGGACGGAT | Reverse primer for KvAP E154A mutation |
| K16F | GCACTGAGCAAAAACGGTTCCTACCCGAGCGGTCATACTTCAATC | Forward primer for KvAP K157A mutation |
| K16R | GTCTTGTTCCGTCGTATTACAGGTGCTCACGCCATAAAACGCAAA | Reverse primer for KvAP K157A mutation |
| K17F | GCAAGCAAAAACGGTTCCTACCCGAGCGGTCATACTTCAATCGGC | Forward primer for KvAP L158A mutation |
| K17R | TTTGTCTTGTTCCGTCGTATTACAGGTGCTCACGCCATAAAACGC | Reverse primer for KvAP L158A mutation |
| K18F | GCAGGTTCCTACCCGAGCGGTCATACTTCAATCGGCTGGGCAACT | Forward primer for KvAP N161A mutation |
| K18R | TTTGCTCAGTTTGTCTTGTTCCGTCGTATTACAGGTGCTCACGCC | Reverse primer for KvAP N161A mutation |
| K19F | GCATCCTACCCGAGCGGTCATACTTCAATCGGCTGGGCAACTGCC | Forward primer for KvAP G162A mutation |
| K19R | GTTTTTGCTCAGTTTGTCTTGTTCCGTCGTATTACAGGTGCTCAC | Reverse primer for KvAP G162A mutation |
| K20F | GCAAGCGGTCATACTTCAATCGGCTGGGCAACTGCCTTGGTTCTG | Forward primer for KvAP P165A mutation |
| K20R | GTAGGAACCGTTTTTGCTCAGTTTGTCTTGTTCCGTCGTATTACA | Reverse primer for KvAP P165A mutation |
| K21F | GCAGGTCATACTTCAATCGGCTGGGCAACTGCCTTGGTTCTGGCG | Forward primer for KvAP S166A mutation |
| K21R | CGGGTAGGAACCGTTTTTGCTCAGTTTGTCTTGTTCCGTCGTATT | Reverse primer for KvAP S166A mutation |
| K22F | GCACATACTTCAATCGGCTGGGCAACTGCCTTGGTTCTGGCGGAG | Forward primer for KvAP G167A mutation |
| K22R | GCTCGGGTAGGAACCGTTTTTGCTCAGTTTGTCTTGTTCCGTCGT | Reverse primer for KvAP G167A mutation |
| K23F | GCAACTTCAATCGGCTGGGCAACTGCCTTGGTTCTGGCGGAGATC | Forward primer for KvAP H168A mutation |
| K23R | ACCGCTCGGGTAGGAACCGTTTTTGCTCAGTTTGTCTTGTTCCGT | Reverse primer for KvAP H168A mutation |
| K24F | GCAGGCTGGGCAACTGCCTTGGTTCTGGCGGAGATCAACCCGCAA | Forward primer for KvAP I171A mutation |
| K24R | TGAAGTATGACCGCTCGGGTAGGAACCGTTTTTGCTCAGTTTGTC | Reverse primer for KvAP I171A mutation |
| K25F | GCAGTGATCTGCGGTTATCATTGGCAGTCCGATGTTGATGCCGCC | Forward primer for KvAP R201A mutation |
| K25R | GCTTTCACCCAGTTCGTAGCCACGTTTCAGGATTTCATTCTGACG | Reverse primer for KvAP R201A mutation |
| K26F | GCATGGCAGTCCGATGTTGATGCCGCCCGTATCGTGGGCTCTGCG | Forward primer for KvAP H207A mutation |
| K26R | ATAACCGCAGATCACACGGCTTTCACCCAGTTCGTAGCCACGTTT | Reverse primer for KvAP H207A mutation |
| K27F | TGTAAGTACATGCGCATCCGTCCGTTTGCGTTTTATGGCGTGAGC | Forward primer for KvAP E134C mutation |
| K27R | Same as K13R | Reverse primer for KvAP E134C mutation |
| K28F | GATAAGTACATGCGCATCCGTCCGTTTGCGTTTTATGGCGTGAGC | Forward primer for KvAP E134D mutation |
| K28R | Same as K13R | Reverse primer for KvAP E134D mutation |
| K29F | TTTAAGTACATGCGCATCCGTCCGTTTGCGTTTTATGGCGTGAGC | Forward primer for KvAP E134F mutation |
| K29R | Same as K13R | Reverse primer for KvAP E134F mutation |
| K30F | GGTAAGTACATGCGCATCCGTCCGTTTGCGTTTTATGGCGTGAGC | Forward primer for KvAP E134G mutation |
| K30R | Same as K13R | Reverse primer for KvAP E134AG mutation |
| K31F | CATAAGTACATGCGCATCCGTCCGTTTGCGTTTTATGGCGTGAGC | Forward primer for KvAP E134H mutation |
| K31R | Same as K13R | Reverse primer for KvAP E134H mutation |
| K32F | ATTAAGTACATGCGCATCCGTCCGTTTGCGTTTTATGGCGTGAGC | Forward primer for KvAP E134I mutation |
| K32R | Same as K13R | Reverse primer for KvAP E134I mutation |
| K33F | AAGAAGTACATGCGCATCCGTCCGTTTGCGTTTTATGGCGTGAGC | Forward primer for KvAP E134K mutation |
| K33R | Same as K13R | Reverse primer for KvAP E134K mutation |
| K34F | CTGAAGTACATGCGCATCCGTCCGTTTGCGTTTTATGGCGTGAGC | Forward primer for KvAP E134L mutation |
| K34R | Same as K13R | Reverse primer for KvAP E134L mutation |
| K35F | ATGAAGTACATGCGCATCCGTCCGTTTGCGTTTTATGGCGTGAGC | Forward primer for KvAP E134M mutation |
| K35R | Same as K13R | Reverse primer for KvAP E134M mutation |
| K36F | AATAAGTACATGCGCATCCGTCCGTTTGCGTTTTATGGCGTGAGC | Forward primer for KvAP E134N mutation |
| K36R | Same as K13R | Reverse primer for KvAP E134N mutation |
| K37F | CCGAAGTACATGCGCATCCGTCCGTTTGCGTTTTATGGCGTGAGC | Forward primer for KvAP E134P mutation |
| K37R | Same as K13R | Reverse primer for KvAP E134P mutation |
| K38F | CAGAAGTACATGCGCATCCGTCCGTTTGCGTTTTATGGCGTGAGC | Forward primer for KvAP E134Q mutation |
| K38R | Same as K13R | Reverse primer for KvAP E134Q mutation |
| K39F | CGTAAGTACATGCGCATCCGTCCGTTTGCGTTTTATGGCGTGAGC | Forward primer for KvAP E134R mutation |
| K39R | Same as K13R | Reverse primer for KvAP E134R mutation |
| K40F | AGCAAGTACATGCGCATCCGTCCGTTTGCGTTTTATGGCGTGAGC | Forward primer for KvAP E134S mutation |
| K40R | Same as K13R | Reverse primer for KvAP E134S mutation |
| K41F | ACCAAGTACATGCGCATCCGTCCGTTTGCGTTTTATGGCGTGAGC | Forward primer for KvAP E134T mutation |
| K41R | Same as K13R | Reverse primer for KvAP E134T mutation |
| K42F | GTTAAGTACATGCGCATCCGTCCGTTTGCGTTTTATGGCGTGAGC | Forward primer for KvAP E134V mutation |
| K42R | Same as K13R | Reverse primer for KvAP E134V mutation |
| K43F | TGGAAGTACATGCGCATCCGTCCGTTTGCGTTTTATGGCGTGAGC | Forward primer for KvAP E134W mutation |
| K43R | Same as K13R | Reverse primer for KvAP E134W mutation |
| K44F | TATAAGTACATGCGCATCCGTCCGTTTGCGTTTTATGGCGTGAGC | Forward primer for KvAP E134Y mutation |
| K44R | Same as K13R | Reverse primer for KvAP E134Y mutation |
| K45F | ACCGGTGTCGCGAACGCGTTCAGCAGCGCGTTCGGCTCCCCGATT | Forward primer for KvAP G91T mutation |
| K45R | CGCAGACAGGTTCGCGTCCTCTGCCGCCAATTTACCGCGCTCGGT | Reverse primer for KvAP G91T mutation |
| K46F | CGTGGTGTCGCGAACGCGTTCAGCAGCGCGTTCGGCTCCCCGATT | Forward primer for KvAP G91R mutation |
| K46R | Same as K45R | Reverse primer for KvAP G91R mutation |
| K47F | GAAGACGCAGGCGATCTCGCGACCCGTGGCGCAAAAGAAAAGTAC | Forward primer for KvAP E122V mutation |
| K47R | AATCATGTTGGTCAGCAATTTGTGCAGCTGCGGAGCGTCCTTCTC | Reverse primer for KvAP E122V mutation |
| K48F | ATTTATTACCTGACCAATGCTCAAGCGATTGACAGCCTGGCTTTG | Forward primer for KvAP L34I mutation |
| K48R | ATCCGGTTTGGTCGTAACATCATTTCCTGGGGGTACTAGAGCCAT | Reverse primer for KvAP L34I mutation |
| K49F | GAAGGCGATCTCGCGACCCGTGGCGCAAAAGAAAAGTACATGCGC | Forward primer for KvAP A124E mutation |
| K49R | GTCCTCAATCATGTTGGTCAGCAATTTGTGCAGCTGCGGAGCGTC | Reverse primer for KvAP A124E mutation |
| K50F | CTGGACGCAGGCGATCTCGCGACCCGTGGCGCAAAAGAAAAGTAC | Forward primer for KvAP E122L mutation |
| K50R | Same as K47R | Reverse primer for KvAP E122L mutation |
| K51F | GATTATTACCTGACCAATGCTCAAGCGATTGACAGCCTGGCTTTG | Forward primer for KvAP L34D mutation |
| K51R | ATCCGGTTTGGTCGTAACATCATTTCCTGGGGGTACTAGAGCCAT | Reverse primer for KvAP L34D mutation |
| K52F | CAGGACGCAGGCGATCTCGCGACCCGTGGCGCAAAAGAAAAGTAC | Forward primer for KvAP E122Q mutation |
| K52R | Same as K47R | Reverse primer for KvAP E122Q mutation |
| K53F | TGGGGTGTCGCGAACGCGTTCAGCAGCGCGTTCGGCTCCCCGATT | Forward primer for KvAP G91W mutation |
| K53R | Same as K45R | Reverse primer for KvAP G91W mutation |
| K54F | TATCTCGCGACCCGTGGCGCAAAAGAAAAGTACATGCGCATCCGT | Forward primer for KvAP D126Y mutation |
| K54R | GCCTGCGTCCTCAATCATGTTGGTCAGCAATTTGTGCAGCTGCGG | Reverse primer for KvAP D126Y mutation |
| K59F | TGGGGTGGTGTCGCGAACGCGTTCAGCAGCGCGTTCGGCTCCCCG | Forward primer for KvAP A90W mutation |
| K59R | AGACAGGTTCGCGTCCTCTGCCGCCAATTTACCGCGCTCGGTGTT | Reverse primer for KvAP A90W mutation |
| K60F | TGTCTCGCGACCCGTGGCGCAAAAGAAAAGTACATGCGCATCCGT | Forward primer for KvAP D126C mutation |
| K60R | GCCTGCGTCCTCAATCATGTTGGTCAGCAATTTGTGCAGCTGCGG | Reverse primer for KvAP D126C mutation |
| K61F | CTGGAGGACGCAGGCAATCTCGCGACCCGTGGCGCAAAAGAAAAG | Forward primer for KvAP I121L mutation |
| K61R | CATGTTGGTCAGCAATTTGTGCAGCTGCGGAGCGTCCTTCTCGGT | Reverse primer for KvAP I121L mutation |
| K62F | AATCTCGCGACCCGTGGCGCAAAAGAAAAGTACATGCGCATCCGT | Forward primer for KvAP D126N mutation |
| K62R | GCCTGCGTCCTCAATCATGTTGGTCAGCAATTTGTGCAGCTGCGG | Reverse primer for KvAP D126N mutation |
| K63F | CCGGACGCAGGCGATCTCGCGACCCGTGGCGCAAAAGAAAAGTAC | Forward primer for KvAP E122P mutation |
| K63R | Same as K45R | Reverse primer for KvAP E122P mutation |
| K64F | TGGGACGCAGGCGATCTCGCGACCCGTGGCGCAAAAGAAAAGTAC | Forward primer for KvAP E122W mutation |
| K64R | Same as K45R | Reverse primer for KvAP E122W mutation |

The nucleotides generating mutation sites were underlined.

## References:

1. Fleishman SJ, Leaver-Fay A, Corn JE et al. RosettaScripts: A Scripting Language Interface to the Rosetta Macromolecular Modeling Suite, PLOS ONE 2011;**6**(6):e20161.

2. Abraham MJ, Murtola T, Schulz R et al. GROMACS: High performance molecular simulations through multi-level parallelism from laptops to supercomputers, SoftwareX 2015;**1-2**:19-25.
